# Indoor Positioning Design for Mobile Phones via Integrating a Single Microphone Sensor and an *H*_2_ Estimator

**DOI:** 10.3390/s23031508

**Published:** 2023-01-29

**Authors:** Yung-Hsiang Chen, Pei-Yu Chang, Yung-Yue Chen

**Affiliations:** 1 Department of Mechanical Engineering, National Pingtung University of Science and Technology, Pingtung 912301, Taiwan; 2 National Chung-Shan Institute of Science and Technology, Taoyuan 32546, Taiwan; 3 Department of Systems and Naval Mechatronic Engineering, National Cheng Kung University, Tainan 701401, Taiwan

**Keywords:** indoor positioning design, sound pressure level, received signal strength, *H*_2_ estimator, energy consumption

## Abstract

An indoor positioning design developed for mobile phones by integrating a single microphone sensor, an *H*_2_ estimator, and tagged sound sources, all with distinct frequencies, is proposed in this investigation. From existing practical experiments, the results summarize a key point for achieving a satisfactory indoor positioning: The estimation accuracy of the instantaneous sound pressure level (SPL) that is inevitably affected by random variations of environmental corruptions dominates the indoor positioning performance. Following this guideline, the proposed *H*_2_ estimation design, accompanied by a sound pressure level model, is developed for effectively mitigating the influences of received signal strength (RSS) variations caused by reverberation, reflection, refraction, etc. From the simulation results and practical tests, the proposed design delivers a highly promising indoor positioning performance: an average positioning RMS error of 0.75 m can be obtained, even under the effects of heavy environmental corruptions.

## 1. Introduction

Indoor positioning designs for mobile communication systems have attracted increasing attention recently due to emergency and security concerns. With the increase in time spent by mobile phone users in indoor environments, a mandatory bill, called Enhanced 911 (E911) was passed by the USA, providing the specific requirements for the indoor positioning accuracy of mobile communication systems. The indoor positioning accuracy requirements of E911 are less than 100 m (67% calls) and 300 m (95% calls) [1]. However, the well-known outdoor positioning system, GPS, is not useful for guaranteeing the accuracy of indoor positioning because of the shielding effect of the positioning signal transmission of satellites, i.e., the positioning ability of GPS is weak in regards to indoor environments such as shopping malls, hypermarkets, office building, etc. A variety of theoretical and available technologies have been proposed in recent decades for achieving the requirements of the indoor positioning [2,3,4,5], based on methods using infrared rays (IR), ultrasound, radio-frequency identification (RFID), wireless local area networks (WLAN), Bluetooth, audible sound, and other technologies. Nevertheless, not all of the above-mentioned methods can be applied to mobile communication systems due to complexity of implementation and the costs of the hardware and software; hence, a new design which is suitable for performing indoor positioning, based on the integration of a single microphone of the mobile communication system and one positioning estimation design. is proposed in this investigation.

Many indoor positioning methods have been proposed in recent years, and an accurate positioning estimation is the common goal of these designs. The earliest design was based on an infrared ray (IR) system [5,6,7,8,9], and until now, it was the most simple and common design for the purpose of indoor positioning. It is possible that the indoor positioning performance of these types of IR-based designs is acceptable in indoor environments which are well-arranged. However, practically, environmental light sources, such as florescent light and sunlight, are always tricky problems which strongly reduce position accuracy [8]. To compensate for the effect of this environment disturbance, several filters have been developed [8,10]. Applications of IR-based indoor positioning designs are constrained due to these interferences, and constructing such a light-based system comes at a high cost. Radio frequency (RF)-based technologies [11,12] are the other designs used for achieving the indoor positioning goal. Categories of RF-based positioning methods are mainly divided into two groups: 1. the radio frequency identification (RFID) method, and 2. the Wireless Local Area Network (WLAN) method. WhereNet is a real-time indoor and outdoor positioning design developed by Zebra Technologies via the RFID method for users who are in intricate environments, such as libraries, offices, etc. [4,13]. Key parts comprising this positioning system include tags, positioning antennas, processors, servers, where ports, and a software algorithm using the differential time of arrival method (TDOA) for calculating the locations of moving tags. 

The major disadvantage of this kind of positioning method is that is necessitates an enormous cost for building numerous infrastructures in the working area. As to the WLAN positioning design, most of WLAN-based algorithms are developed via adopting the received signal strength of the WLAN signals. Generally speaking, WLAN-based designs possess a low-cost feature due to the popularity of WLAN infrastructures in indoor environments. By building on this advantage, a RADAR positioning system has been developed by a Microsoft research group, combining received signal strength detection with the triangulation positioning method. Unfortunately, the received signal strength of WLAN is naturally and inevitably affected by various environmental uncertainties, such as the multipath effect, the no line-of-sight effect [14,15,16,17], etc.; hence, some auxiliary designs are proposed to reduce the effect of environmental uncertainties [15,18,19], e.g., a radio map using the fingerprinting method, or the approximation design of a specific environment using the fuzzy logic approach. Fingerprinting techniques work well when the stored information of WLAN access points (APs) increase significantly, i.e., more APs are needed [20]. Algorithms based on sound detection provide another potential method for indoor positioning designs. The Active Bat system was developed by AT&T Cambridge by mimicking the navigation behavior of bats [21]. 

For improving the accuracy of the Active Bat system, a new design called the Cricket system combines the overall design of the Active Bat system with an extra RF method [22,23]. For the above developments, multi-sensors and ultrasound designs are adopted. Daredevil, developed by Microsoft, uses audible sound sources, providing an indoor positioning ability for mobile phones with at least two embedded microphones [4]. In the work in [14], the mobile phone is used as an emitter, and it collocates with the Wi-Fi network to achieve higher accuracy. A contrasting design, in which the handheld devices are arranged as receivers for some predefined emitters, is another popular design because mobile phone users always need the real-time display of the mobile phones’ monitors [24,25]. The positioning algorithm based on TOA or TDOA methods requires that multi-sensors be used, i.e., the total costs will be higher than those using the single sensor design; besides, the TOA or TDOA methods are degraded by four main factors: 1. background noise, 2. the multi-path effect [26,27], 3. non line-of-sight propagation [28,29], and 4. mobile synchronization recovery [30,31]. 

Therefore, an indoor positioning algorithm which can be easily implemented in a mobile phone using just one microphone is proposed. Unlike in the above design, in which mobile phones were set up as emitters, in this study, the mobile phone’s microphone is arranged as the receiver of tagged sound sources using distinct frequencies. Four tagged sound sources with distinct frequencies are broadcasted from speakers placed in the corners of an indoor space, and for reducing the total cost of the hardware, only one mobile phone microphone is used to collect messages of different tagged sound sources. Regarding the development of the methodology, one novel indoor positioning method combining the received signal strength (RSS) method, the fast Fourier transform (FFT) method, *H*_2_ estimation design, and the intersection of circles method is proposed. In this proposed method, RSS is used to calculate the strength of each tagged sound source, FFT is adopted to analyze the spectrum of the collected data of tagged sound sources, the *H*_2_ estimation design is used to purify the noisy tagged sound source, and output denoised sound pressure level (SPL), and an accurately estimated position of the user in an indoor environment can then be solved by the intersection of circles method.

## 2. The Proposed Indoor Positioning Algorithm

**Figure 1:**
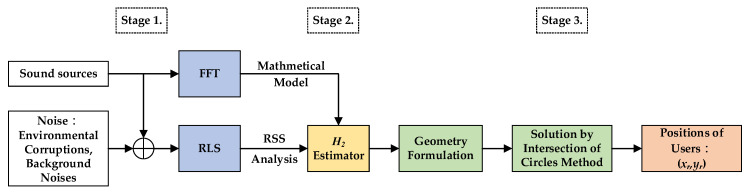
Flow chart of the proposed indoor positioning algorithm.

The overall schematic of the proposed indoor positioning design is shown as Figure 1. In this investigation, this procedure is separated into three stages. A RSS analysis of the first stage is used to verify the pressure of the received signals measured by the single microphone of the mobile phones; additionally, in this stage, one system identification method—recursive least square (RLS)—and the famous power spectrum analysis tool—FFT—are utilized to simulate the walk behavior of users and make SPL selections regarding which two tagged sound sources will be adopted. In the second stage, one novel estimation design possesses an effective reduction ability regarding environmental corruptions. A set of purified SPLs can be obtained by using this proposed estimation design, and the first two of these four SPLs, which exhibit the strongest intensities, will be used as inputs of geometry equations in the third stages. Based on the purified SPLs, four geometry equations, which are functions of the users’ indoor position $\left(x_{r}(k),y_{r}(k)\right)$, can be easily determined from the relationship between tagged sound sources and the microphone of the user’s mobile phone. The users’ indoor position $\left(x_{r}(k),y_{r}(k)\right)$ can be further solved by the intersection of circles method in the third stage.

### 2.1. Data Acquisition and RSS Analysis

In the following, a detailed mathematical expression for these three stages will be derived. In the first stage, the received raw data of the four tagged sound sources are as follows: $S_{n}$, for n=1 to 4, with distinct tagged frequencies, should be identified. As to the choice of these two tagged sound sources, there are two steps arranged previously: calculations of magnitudes and frequencies. For identifying the magnitude and frequency, FFT and RSS analysis are used. The tagged frequency of each sound source cannot be higher than 20 kHz due to the physical limitation of the standard microphone used, and the sampling frequency of the mobile phone is 8 kHz; hence, tag frequencies of the sound sources are selected as 15.5 kHz, 16.5 kHz, 17.5 kHz, and 18.5 kHz, respectively. For separating the four tagged sound sources, a specific frequency tag $f_{Sn}$ is assigned for each of the four tagged sound sources, as follows:

(1) $$O_{n}:\left(14000+1000n\right)Hz<f_{Sn}<\left(15000+1000n\right)Hz,\;\mathrm{for}\;n=1\;\mathrm{to}\;4$$

Denote the received signals of the tagged sound sources $S_{n}$ as $z_{Sn}$, for n=1 to 4, which contain all surrounding sounds. In this stage, two tagged sound candidates will be selected based on the messages of magnitudes and frequencies of these four tagged sound sources. For the purpose of calculating magnitudes (dB) and frequencies (Hz) of the tagged sound sources, the famous power spectrum method FFT is used. According to the definition of FFT, the magnitude for these four distinct frequencies can be expressed as:

(2) $$F_{Sn}\left(k\right)=\mathrm{FFT}\left\{z_{Sn}\left(k\right)\right\},\;\mathrm{for}\;n=1\;\mathrm{to}\;4$$

A mean value magnitude adopted for calculation of the intensities of the tagged sound sources is defined as the following:

(3) $$C_{Sn}=\frac{1}{B}\sum_{\kappa =0}^{B}F_{Sn}\left(k\right),\;\mathrm{for}\;n=1\;\mathrm{to}\;4$$

where B is the number of bins. Equations (2) and (3) will be utilized to identify the received tagged sound sources. 

### 2.2. Indoor Positioning Algorithm

**Figure 2:**
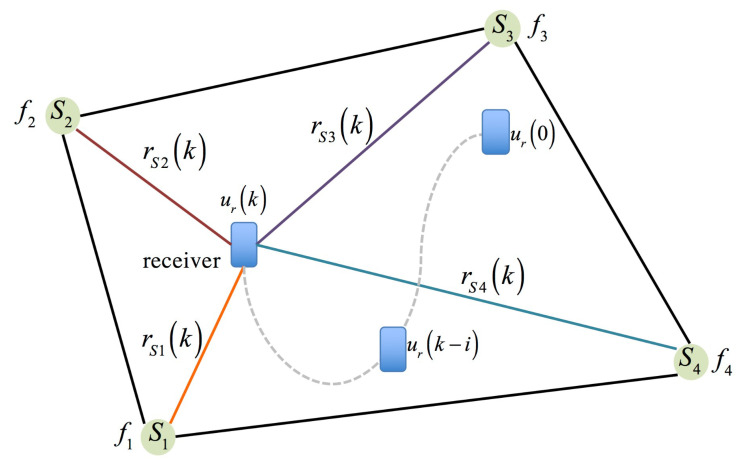
Geometric relationship between the carried receivers with respect to tagged sound sources.

The geometry relationship between the carried receiver and distinct tagged sound sources is illustrated in Figure 2. In Figure 2, the carried receiver is set up as the mobile phone and is initialized as $u_{r}\left(0\right)=\left\{x_{r}\left(0\right),y_{r}\left(0\right)\right\}$. Suppose at least four tagged sound sources, with distinct frequencies, are placed in four corners of an indoor space, and their coordinates are represented as $\left(x_{1},y_{1}\right)$, $\left(x_{2},y_{2}\right)$, $\left(x_{3},y_{3}\right)$, and $\left(x_{4},y_{4}\right)$.

Figure 2 is an irregular-shaped indoor plane, and there are four tagged sound sources placed in four corners. Tagged sound sources are denoted as $S_{n}$, for n=1 to 4, and tag frequencies of these sound sources are set up as $f_{Sn}$. As to the initial locations of the tagged sound sources, they are placed in $\left(x_{n},y_{n}\right)$ of the test indoor plane. The moving data of the carried receiver in every time instant is defined as:

(4) $$u_{r}=\left\{\left(x_{r}(k),y_{r}(k)\right)\in R^{2}\right\},\;\mathrm{for}\;1\leq k\leq N$$

where N is number of indoor positioning iterations.

In the following, the transformation of SPLs and the relative distances will be detailed. 

Data collection of SPLs: The carried receiver collects four different SPL values from the tagged sound sources at each sampling time, which can be expressed as a set *L_Sn_*:

(5) $$L_{Sn}=\left\{l_{Sn}\left(k\right)\in R^{1}\right\},\;\mathrm{for}\;n=1\;\mathrm{to}\;4,\;\mathrm{and}\;1\leq k\leq N$$

where $l_{Sn}$ is the measured SPL of $S_{n}$ with the tag frequency $f_{Sn}$.

Relative distances between each tagged sound source and the receiver can be expressed as the following four sets.

(6) $$R_{Sn}=\left\{r_{Sn}\left(k\right)\in R^{1}\right\},\;\mathrm{for}\;n=1\;\mathrm{to}\;4,\;\mathrm{and}\;1\leq k\leq N$$

where $r_{Sn}\left(k\right)$ is the relative distance from the positions of tagged sound sources $S_{n}$ to the receiver. Based on Equations (5) and (6), the instantaneous relative distance between each tagged sound source and the receiver can be calculated from the SPL difference $l_{Sn}\left(k\right)$ and $l_{Sn}\left(k-1\right)$ as

(7) $$r_{Sn}\left(k\right)=r_{Sn}\left(k-1\right)/10^{\frac{l_{Sn}\left(k\right)-l_{Sn}\left(k-1\right)}{20}}+{\left(\frac{R_{C}+16\pi r_{2}^{2}}{R_{C}+16\pi r_{1}^{2}}\right)}^{\frac{1}{2}},\;\mathrm{for}\;n=1\;\mathrm{to}\;4,\;\mathrm{and}\;1\leq k\leq N$$

where $R_{c}$ is the uncertainty room constant. Due to the fact that $R_{c}$ cannot be measured in prior, it is regarded as a modeling uncertainty. Based on this, the corrupted relative distance $r_{Sn}\left(k\right)$ can be further expressed as below:

(8) $$r_{Sn}\left(k\right)=r_{Sn}\left(k-1\right)/10^{\frac{l_{Sn}\left(k\right)-l_{Sn}\left(k-1\right)}{20}}+w\left(k\right),\;\mathrm{for}\;n=1\;\mathrm{to}\;4,\;\mathrm{and}\;1\leq k\leq N$$

where initial values of $r_{Sn}\left(k\right)$ and $l_{Sn}\left(k\right)$ are $r_{Sn}\left(0\right)$ and $l_{Sn}\left(0\right)$, respectively, and $w\left(k\right)={\left(\frac{R_{C}+16\pi r_{2}^{2}}{R_{C}+16\pi r_{1}^{2}}\right)}^{\frac{1}{2}}$.

The next section will introduce the method of extracting the noiseless SPLs with distinct tagged frequencies and derive the geometry mathematical formulation for finding the instantons positions of the mobile phone users. 

#### 2.2.1. Geometric Mathematical Formulation

**Figure 3:**
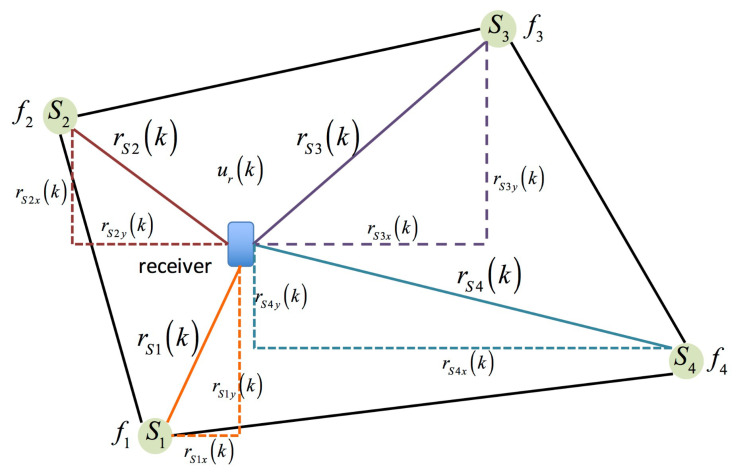
The geometric relationship between the tagged sound sources and the receiver in an indoor plane.

In Figure 3, there are four right angle triangles inside a quadrilateral, and each of them describes the relationship between the receiver and each tagged sound sources. 

Based on Figure 3, four equations are derived, and these equations express the geometric relationships between the receiver and the tagged sound sources in an indoor plane.

(9) $${\left(x_{1}-x_{r}\left(k\right)\right)}^{2}+{\left(y_{1}-y_{r}\left(k\right)\right)}^{2}=r_{S1}{\left(k\right)}^{2}$$

(10) $${\left(x_{2}-x_{r}\left(k\right)\right)}^{2}+{\left(y_{2}-y_{r}\left(k\right)\right)}^{2}=r_{S2}{\left(k\right)}^{2}$$

(11) $${\left(x_{3}-x_{r}\left(k\right)\right)}^{2}+{\left(y_{3}-y_{r}\left(k\right)\right)}^{2}=r_{S3}{\left(k\right)}^{2}$$

(12) $${\left(x_{4}-x_{r}\left(k\right)\right)}^{2}+{\left(y_{4}-y_{r}\left(k\right)\right)}^{2}=r_{S4}{\left(k\right)}^{2}$$

where $\left(x_{1},y_{1}\right)$,$\left(x_{2},y_{2}\right)$,$\left(x_{3},y_{3}\right)$, and $\left(x_{4},y_{4}\right)$ are the coordinates of the tagged sound sources, and these positions are fixed and known. $\left(x_{r}(k),y_{r}(k)\right)$ is the coordinate of the receiver at time instant k. $r_{Sn}\left(k\right)$, n=1 to 4 are the relative distances of the receiver to the tagged sound sources $S_{n}$ at time instant k and can be calculated from Equation (8).

From the above mathematical formulation for the geometric relationship between the tagged sound sources and the receiver, four equations are obtained. However, only two of these four equations will be utilized to solve the two unknowns:$\left(x_{r}(k),y_{r}(k)\right)$, and two of these four equations—Equations (9)–(12)—will be selected according to the measured SPLs by using Equations (2) and (3). The criterion for selecting the two equations is $l_{Max}=Max\;L_{Sn}$ and $l_{\mathrm{Sec}}=Max\left\{L_{Sn}-Max\;L_{Sn}\right\}$, e.g., if the intensity sequence of the measured SPLs is in sequence: $l_{S2}>l_{S3}>l_{S1}>l_{S4}$ based on Equations (3), (10) and (11), the following will then be selected and combined as a set of binary quadratic equations as:

(13) $${\left(x_{2}-x_{r}\left(k\right)\right)}^{2}+{\left(y_{2}-y_{r}\left(k\right)\right)}^{2}=r_{S2}{\left(k\right)}^{2}$$

(14) $${\left(x_{3}-x_{r}\left(k\right)\right)}^{2}+{\left(y_{3}-y_{r}\left(k\right)\right)}^{2}=r_{S3}{\left(k\right)}^{2}$$

#### 2.2.2. Solution by Intersection of Two Circles Method

For solving the solution $\left(x_{r}(k),y_{r}(k)\right)$ of the binary quadratic equations, the intersection of two circles method is applied. The reason for using this method is that it offers calculation convenience and a low computational burden for calculators of mobile phones.

**Figure 4:**
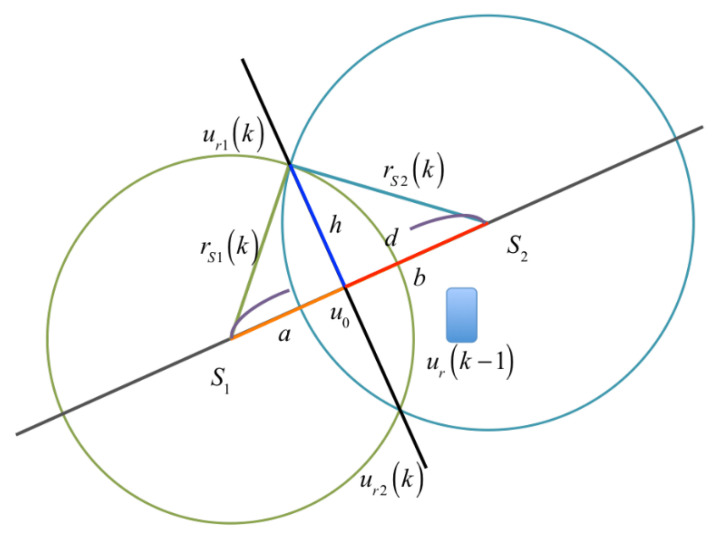
Intersection of two circles.

In Figure 4, $S_{1}$ and $S_{2}$ are tagged sound sources which are selected from Figure 3. The coordinates are $\left(x_{1},y_{1}\right)$ and $\left(x_{2},y_{2}\right)$, respectively. Two overlapped circles can be illustrated when a radius $r_{S1}\left(k\right)$ and $r_{S2}\left(k\right)$, which are the relative distance between the receiver and the tagged sound source, are assigned for tagged sound sources $S_{1}$ and $S_{2}$, respectively. Suppose $r_{S1}\left(k\right)$ and $r_{S2}\left(k\right)$ are large enough to intersect with each other. Two intersect point, $u_{r1}\left(k\right)$ and $u_{r2}\left(k\right)$, can be obtained, as shown in Figure 4. Theoretically, one of these two points, ($u_{r1}\left(k\right)$ and $u_{r2}\left(k\right)$), would be the solution of the corresponding binary quadratic equations.

Denoting the distance $\bar{S_{1}S_{2}}$ as d, it can be expressed as:

(15) $$d=a+b=\sqrt{{\left(x_{2}-x_{1}\right)}^{2}+{\left(y_{2}-y_{1}\right)}^{2}}$$

where a is $\bar{S_{1}u_{0}}$ and b is $\bar{S_{2}u_{0}}$.

There are two equations obtained from triangles $\Delta S_{1}u_{r1}u_{0}$ and $\Delta S_{2}u_{r1}u_{0}$ as

(16) $$\begin{array}{l} a^{2}+h^{2}=r_{S1}{\left(k\right)}^{2}\\ b^{2}+h^{2}=r_{S2}{\left(k\right)}^{2} \end{array}$$

Using d=a+b, the variable a can be solved as:

(17) $$a=\left(r_{S1}{\left(k\right)}^{2}-r_{S2}{\left(k\right)}^{2}+d^{2}\right)/\left(2d\right)$$

The solution of the point $u_{0}\left(x_{u_{0}},y_{u_{0}}\right)$ can be obtained

(18) $$\left(x_{u_{0}},y_{u_{0}}\right)=\left(x_{1},y_{1}\right)+\frac{a}{d}\left(x_{2}-x_{1},y_{2}-y_{1}\right)$$

Substituting a into Equation (16) to solve h, the coordinate of the intersection point $u_{r}\left(k\right)=\left(x_{r}\left(k\right),y_{r}\left(k\right)\right)$ can be obtained as below:

(19) $$\begin{array}{l} x_{r}\left(k\right)=x_{u_{0}}\pm \frac{h}{d}\left(y_{2}-y_{1}\right)\\ y_{r}\left(k\right)=y_{u_{0}}\pm \frac{h}{d}\left(x_{2}-x_{1}\right) \end{array}$$

(20) $$\begin{array}{l} x_{r}\left(k\right)=x_{1}+\frac{a}{d}\left(x_{2}-x_{1}\right)\pm \frac{\sqrt{r_{S1}{\left(k\right)}^{2}-a^{2}}}{d}\left(y_{2}-y_{1}\right)\\ y_{r}\left(k\right)=y_{1}+\frac{a}{d}\left(y_{2}-y_{1}\right)\pm \frac{\sqrt{r_{S1}{\left(k\right)}^{2}-a^{2}}}{d}\left(x_{2}-x_{1}\right) \end{array}$$

In Equation (20), two position solutions of the receiver are obtained simultaneously, but only one of them is the correct answer. There is a simple method to determine whether it is the correct one: the correct solution $u_{r}\left(k\right)$ must be inside the indoor plane.

However, there are some special cases in which two solutions of Equation (20) are both inside the quadrilateral $S_{1}S_{2}S_{3}S_{4}$. One judgment is proposed for the reasonable selection of these special cases by considering the moving velocity of the users. Normally, a moving velocity of 10 m/s is the maximum limitation for most of users. To calculate the moving velocity by the difference of current positioning solution $u_{r}\left(k\right)$ and the previous solution $u_{r}\left(k-1\right)$, one checking condition can found for the selection of the correct solution as:

(21) Δur(k)=ur(k)−ur(k−1)t≤10ms

where t is the sampling time of the system.

The solution of Equation (20) based on the accurate measurement of SPLs $l_{Sn}\left(k\right)$, is n=1 to 4. Naturally, environmental corruptions, such as reverberation, interference, etc., are always contained in the measurement process of SPLs, hence the SPLs $l_{Sn}\left(k\right)$ should be denoised before calculating Equations (8) and (20). For treating this noise reduction problem regarding the corrupted SPLs $l_{Sn}\left(k\right)$, an estimation design for effectively removing the environmental corruptions and purifying the SPLs $l_{Sn}\left(k\right)$ is proposed. In the following, a systematic estimation design, combining RLS system modeling and a steady-state optimal estimator, is developed for denoising the corrupted SPLs. 

### 2.3. System Modeling

Before estimating the correct SPLs via adopting an optimal estimation method, each of the received SPLs should first be mathematically modeled. Suppose a set of the measured SPLs can be described as:

(22) $$L_{all}=\left[l_{S1},\ldots ,l_{S4}\right]$$

where $L_{all}$ is the set of all measured SPL data, and $l_{Sn}$, for n=1 to 4 is the set of measured SPL data from the tagged sound sources.

The mathematical models of the received SPLs can be expressed as the following white-noise driven difference equation:

(23) $$l_{Sn}\left(k\right)=\sum_{i=1}^{m}a_{Sni}l_{Sn}\left(k-i\right)+w_{Sn}\left(k\right),\;\mathrm{for}\;n=1,\ldots ,4$$

(24) $$Z_{Sn}\left(k\right)=l_{Sn}\left(k\right)+v_{Sn}\left(k\right)$$

where $Z_{Sn}\left(k\right)$ is the noisy measurement output of the tagged sound source n. Moreover, $w_{Sn}\left(k\right)$ and $v_{Sn}\left(k\right)$ are Gaussian white noise, with a zero mean and which are uncorrelated with $l_{Sn}\left(k\right)$. $a_{Sni}$, for *i* = 1, …, *m* are identifiable system parameters of the tagged sound source n, and m is the system order.

A regressive form is used to express the difference equation in Equation (23):

(25) $$l_{Sn}\left(k\right)=\Psi_{Sn}{\left(k\right)}^{T}{\hat{\lambda }}_{Sn}\left(k\right)+w_{Sn}\left(k\right)$$

where $\Psi_{Sn}\left(k\right)={\left[l_{Sn}\left(k-1\right)\;\cdots \;l_{Sn}\left(k-m\right)\right]}^{T}$ is the regression vector which contains the measured SPL data, and ${\hat{\lambda }}_{Sn}\left(k\right)=\left[a_{Sn1}\;\cdots \;a_{Snm}\right]$ is the parameter vector.

Equation (25) can be further formulated as a state space form, as follows:

(26) $$L_{Sn}\left(k+1\right)=\Psi_{Sn}L_{Sn}\left(k\right)+\Lambda w_{Sn}\left(k\right)$$

(27) $$Z_{Sn}\left(k\right)=\Omega L_{Sn}\left(k\right)+\Pi v_{Sn}\left(k\right)$$

where $\Psi_{Sn}=\left[\begin{array}{ccccc} a_{Sn1} & a_{Sn2} & \cdots & a_{Snm-1} & a_{Snm}\\ 1 & 0 & \cdots & \cdots & 0\\ 0 & \ddots & \ddots & \ddots & \vdots \\ \vdots & \ddots & \ddots & \ddots & \vdots \\ 0 & \cdots & 0 & 1 & 0 \end{array}\right]\in {ℜ}^{m\times m},\Lambda =\left[\begin{array}{c} 1\\ 0\\ 0\\ \vdots \\ 0 \end{array}\right]\in {ℜ}^{m\times 1},\Omega ={\left[\begin{array}{c} 1\\ 0\\ 0\\ \vdots \\ 0 \end{array}\right]}^{T}\in {ℜ}^{1\times m}$, Π=1 and $L_{Sn}\left(k\right)={\left[l_{Sn}\left(k\right)\;l_{Sn}\left(k-1\right)\;\cdots \;l_{Sn}\left(k-m+2\right)\;l_{Sn}\left(k-m+1\right)\right]}^{T}$ is the state vector, and $w_{Sn}\left(k\right)$ and $v_{Sn}\left(k\right)$ are white noises.

**Remark** **1.** *The system order m is a user decided parameter. Theoretically, a more accurate model can be found by increasing this system order. Surely, a high order system needs more calculation time and storage memory. There exists a tradeoff when selecting the system parameter m. System parameters *$a_{Sni}$, for i=*1 to m can be optimally calculated by several identification methods, such as the recursive least squares method (RLS), the stochastic subspace identification method (SSI), and the system realization using information matrix (SRIM). In this investigation, the system parameters* $a_{Sni}$*in Equation (23) will be optimally determined by the RLS method.*

The RLS algorithm is often used for searching the optimal parameters of systems with a set of unknown parameters by using the input and output measured raw data. The standard identification process of this algorithm can be expressed as follows:

(28) $$\varepsilon_{Sn}\left(k\right)=Z_{Sn}\left(k\right)-\Psi_{Sn}{\left(k\right)}^{T}{\hat{\lambda }}_{Sn}\left(k-1\right)$$

(29) $${\bar{Q}}_{Sn}\left(k\right)=\frac{1}{\gamma }\left[{\bar{Q}}_{Sn}\left(k-1\right)-\frac{{\bar{Q}}_{Sn}\left(k-1\right)\Psi_{Sn}\left(k\right){\hat{\lambda }}_{Sn}{\left(k\right)}^{T}{\bar{Q}}_{Sn}\left(k-1\right)}{\gamma +\Psi_{Sn}{\left(k\right)}^{T}{\bar{Q}}_{Sn}\left(k-1\right){\hat{\lambda }}_{Sn}\left(k\right)}\right]$$

(30) $${\hat{\lambda }}_{Sn}\left(k\right)={\hat{\lambda }}_{Sn}\left(k-1\right)+{\bar{Q}}_{Sn}\left(k\right)\Psi_{Sn}\left(k\right)\varepsilon_{Sn}\left(k\right)$$

where ${\bar{Q}}_{Sn}\left(k\right)$ is the estimation of the coefficient covariance at time instant k, ${\hat{\lambda }}_{Sn}\left(k\right)$ is the identified parameter, $\Psi_{Sn}\left(k\right)$ is the input data, $\varepsilon_{Sn}\left(k\right)$ is the prediction error, $Z_{Sn}\left(k\right)$ is the measurement output, and γ is the forgetting factor. The range of forgetting factor γ is usually given within 0.95 to 1. 

### 2.4. H_2_ Estimation Design

Based on the identified system parameters ${\hat{\lambda }}_{Sn}\left(k\right)$ in Equation (30), an *H*_2_ estimator is proposed for eliminating the influence of the environmental corruptions.

Equations (26) and (27) represent the state-space system of measured SPLs, and the purified SPLs $h_{Sn}\left(k\right)$ can be reconstructed as:

(31) $$h_{Sn}\left(k\right)=JL_{Sn}\left(k\right)$$

where J is a constant matrix that is set up to draw out the purified SPL $h_{Sn}\left(k\right)$ from state vector $L_{Sn}\left(k\right)$. The designed target is to hunt for the estimation ${\hat{L}}_{Sn}\left(k\right)$ from the measured SPL $Z_{Sn}\left(k\right)$, which is corrupted by environmental noises; hence, the state estimator for purifying the corrupted SPL is formulated as the following:

(32) $$\begin{array}{l} {\hat{L}}_{Sn}\left(k+1\right)=\Psi_{Sn}{\hat{L}}_{Sn}\left(k\right)+G_{Sn}\left[Z_{Sn}\left(k\right)-\Omega {\hat{L}}_{Sn}\left(k\right)\right]\\ {\hat{h}}_{Sn}\left(k\right)=J{\hat{L}}_{Sn}\left(k\right) \end{array}$$

where $G_{Sn}\in {ℜ}^{m\times 1}$ is the designed estimation gain in a steady state.

Define the estimation error between purified SPL signal and estimation signal as follows:

(33) $$\begin{array}{ll} {\tilde{e}}_{Sn}\left(k\right) & =h_{Sn}\left(k\right)-{\hat{h}}_{Sn}\left(k\right)\\ & =JL_{Sn}\left(k\right)-J{\hat{L}}_{Sn}\left(k\right)\\ & =J{\tilde{L}}_{Sn}\left(k\right) \end{array}$$

where ${\tilde{L}}_{Sn}\left(k\right)=L_{Sn}\left(k\right)-{\hat{L}}_{Sn}\left(k\right)$

The performance index of the *H*_2_ estimation design of the indoor positioning problem can be expressed by using the mean square error of estimation error ${\tilde{e}}_{Sn}\left(k\right)$ as:

(34) $$\begin{array}{ll} X_{Sn} & =E\left\{{\tilde{e}}_{Sn}\left(k+1\right){\tilde{e}}_{Sn}{\left(k+1\right)}^{T}\right\}\\ & =E\left\{J{\tilde{L}}_{Sn}\left(k+1\right){\tilde{L}}_{Sn}{\left(k+1\right)}^{T}J^{T}\right\} \end{array}$$

where ${\tilde{e}}_{Sn}\left(k+1\right)=J{\tilde{L}}_{Sn}\left(k+1\right)$

Furthermore, Equation (34) can be presented as:

(35) $$\begin{array}{ll} X_{Sn} & =E\left\{tr\left(J{\tilde{L}}_{Sn}\left(k+1\right){\tilde{L}}_{Sn}{\left(k+1\right)}^{T}J^{T}\right)\right\}\\ & =tr\left(JE\left\{{\tilde{L}}_{Sn}\left(k+1\right){\tilde{L}}_{Sn}{\left(k+1\right)}^{T}\right\}J^{T}\right) \end{array}$$

From Equation (26), the estimation error ${\tilde{L}}_{Sn}\left(k+1\right)$ at a steady state can be described as:

(36) $$\begin{array}{ll} {\tilde{L}}_{Sn}\left(k+1\right) & =L_{Sn}\left(k+1\right)-{\hat{L}}_{Sn}\left(k+1\right)\\ & =\Psi_{Sn}L_{Sn}\left(k\right)+\Lambda w_{Sn}\left(k\right)\\ & \;\;\;\;\;-\left\{\Psi_{Sn}{\hat{L}}_{Sn}\left(k\right)+G_{Sn}\left[Z_{Sn}\left(k\right)-\Omega {\hat{L}}_{Sn}\left(k\right)\right]\right\}\\ & =\Psi_{Sn}\left(L_{Sn}\left(k\right)-{\hat{L}}_{Sn}\left(k\right)\right)+\Lambda w_{Sn}\left(k\right)\\ & \;\;\;\;\;-G_{Sn}\left[\Omega L_{Sn}\left(k\right)+\Pi v_{Sn}\left(k\right)-\Omega {\hat{L}}_{Sn}\left(k\right)\right]\\ & =\Psi_{Sn}{\tilde{L}}_{Sn}\left(k\right)+\Lambda w_{Sn}\left(k\right)-G_{Sn}\left[\Omega {\tilde{L}}_{Sn}\left(k\right)+\Pi v_{Sn}\left(k\right)\right]\\ & =\left(\Psi_{Sn}-G_{Sn}\Omega \right){\tilde{L}}_{Sn}\left(k\right)+\Lambda w_{Sn}\left(k\right)-G_{Sn}\Pi v_{Sn}\left(k\right) \end{array}$$

After some mathematical derivations, the *H*_2_ estimation design for background noise reduction of the indoor positioning design could be summarized as the following Theorem 1.

**Theorem** **1.** *An H_2_ steady state estimator for the indoor positioning problem can be constructed if a positive-definite matrix* $E_{Sn}=E_{Sn}^{T}$* can be found such that the following LMIs hold*

(37) $$\left[\begin{array}{cccc} E_{Sn} & E_{Sn}\Lambda & D_{Sn}\Pi & \left(E_{Sn}\Psi_{Sn}-D_{Sn}\Omega \right)\\ \Lambda^{T}E_{Sn} & I & 0 & 0\\ \Pi^{T}D_{Sn}^{T} & 0 & I & 0\\ {\left(E_{Sn}\Psi_{Sn}-D_{Sn}\Omega \right)}^{T} & 0 & 0 & E_{Sn} \end{array}\right]>0\;$$

*where* $D_{Sn}=E_{Sn}G_{Sn}$ *, and the steady state covariance of the estimation error is bounded by*

(38) $$E\left\{{\tilde{e}}_{Sn}\left(k+1\right){\tilde{e}}_{Sn}{\left(k+1\right)}^{T}\right\}<tr\left(JE_{Sn}^{-1}J^{T}\right)$$

**Remark** **2.** *Proof of Theorem 1 is given in*Appendix A.

From Equation (37), $D_{Sn}$ and $E_{Sn}$ can be computationally calculated. Based on these two parameters, the estimation gain of the *H*_2_ estimation design can be obtained by using $G_{Sn}=E_{Sn}^{-1}D_{Sn}$. Additionally, by substituting $G_{Sn}$ into Equation (32), the *H*_2_ estimator can be constructed.

The process of constructing the proposed *H*_2_ estimation design is summarized in the following steps:

Step 1. Given the $R_{Sn}$, $T_{Sn}$ as identity matrices, and J as a constant matrix based on the extraction of desired state variables.
Step 2. Solve the LMI form of Equation (37) for obtaining the positive matrix $D_{Sn}$ and $E_{2Sn}$.
Step 3. Calculate the estimation gain $G_{Sn}$ based on solutions of $E_{Sn}$ and $D_{Sn}$ in Step 2.
Step 4. Substituting the estimation gain $G_{Sn}$ into Equation (32), the $H_{2}$ estimation design can be constructed as Equation (39).

(39) $$\begin{array}{c} {\hat{L}}_{Sn}\left(k+1\right)=\Psi_{Sn}{\hat{L}}_{Sn}\left(k\right)+G_{Sn}\left[Z_{Sn}\left(k\right)-\Omega {\hat{L}}_{Sn}\left(k\right)\right]\\ {\hat{h}}_{Sn}\left(k\right)=J{\hat{L}}_{Sn}\left(k\right) \end{array}$$

## 3. Verification of Indoor Positioning Performance

To verify the proposed indoor positioning system, simulation results and practical tests will be discussed and compared. Before discussing the positioning performance of this proposed design, the environmental settings of the hardware and software used will be detailed first. Next, the simulation results, which contain one scenario, will be discussed and analyzed. The practical implementation and testing of this proposed design will be verified after the simulation process. Finally, the comparisons of the simulation results and the practical tests will be discussed. 

### 3.1. Testing Environment Setting

#### 3.1.1. Arrangements of Hardware

**Figure 5:**
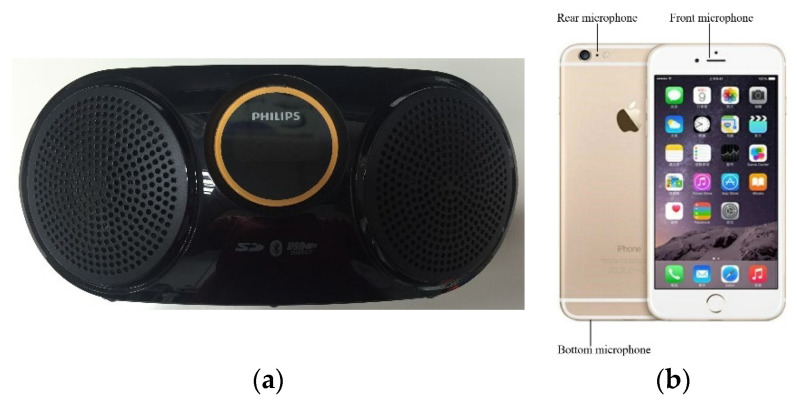
The used devices: (**a**) Wireless speaker (Philips AT10). (**b**) iPhone6.

Hardware adopted for the indoor positioning verification of this proposed design comprises four speakers: Philips AT10, for broadcasting tagged sound sources, one iPhone6 microphone used as a receiver, and a dual core CPU of an iPhone 6 adopted as the calculator of the proposed indoor positioning algorithm. Specifications of the Philips AT10 and the iPhone 6 are displayed in Figure 5 and are listed in Table 1 and Table 2.

#### 3.1.2. Software Design

After choosing the hardware above, the distinct tag frequencies can be calculated by the FFT method using the measured raw data of the receiver. The analog to digital resolution of iPhone 6 is 16 bits; hence, the measured analog sound pressure of these four tagged sound sources can be presented with 2048 bits.

Distinct tagged frequencies are selected from the following frequency spans:

(40) $$\begin{array}{l} 15\;kHz<f_{S1}<16\;kHz\\ 16\;kHz<f_{S2}<17\;kHz\\ 17\;kHz<f_{S3}<18\;kHz\\ 18\;kHz<f_{S4}<19\;kHz \end{array}$$

The setting parameters for the distinct tagged sound source are shown in Table 3, including tag frequencies, relative distances, and SPL values, respectively. These values are defined as the initial values and are fixed.

For analyzing the effects of the inevitable environmental corruptions which infiltrate to the located tagged sound sources. Two background sounds: 1. one recorded frame of a really noisy pub and 2. a song named: Free loops are used as the environmental corruptions for verifying the robust property of this proposed indoor positioning design. 

### 3.2. Test Results

#### 3.2.1. The Configuration for Practical Results

**Figure 6:**
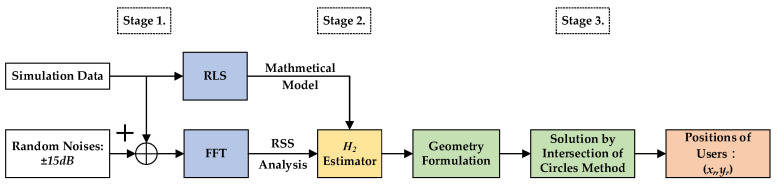
The overall flow chart of these proposed indoor positioning designs.

For simulations, a flow chart with three stages each playing specific roles in the indoor positioning process, is illustrated in Figure 6. The corruptions of environments, such as reverberant effects and environmental uncertainties, are set up as random noises, with the magnitude 15 dB for mimicking the practical situations.

The parameters for the proposed *H*_2_ estimator in Equation (39) are given in Table 4, and for conserving computational power, the system order *m* for the SPL model in Equation (23) is selected as 15. The steady state values ${\hat{\lambda }}_{Sn}\left(k\right)$, $G_{Sn}$, and $E_{Sn}$, for *n*=1 to 4, of SPL models with respect to tagged sound sources S1 to S4 are listed in Table 4. For saving space, only ${\hat{\lambda }}_{S1}\left(k\right)$, $G_{S1}$, and $E_{S1}$ of the tagged sound source S1 are listed in Table 4. 

#### 3.2.2. Testing Scenario

**Figure 7:**
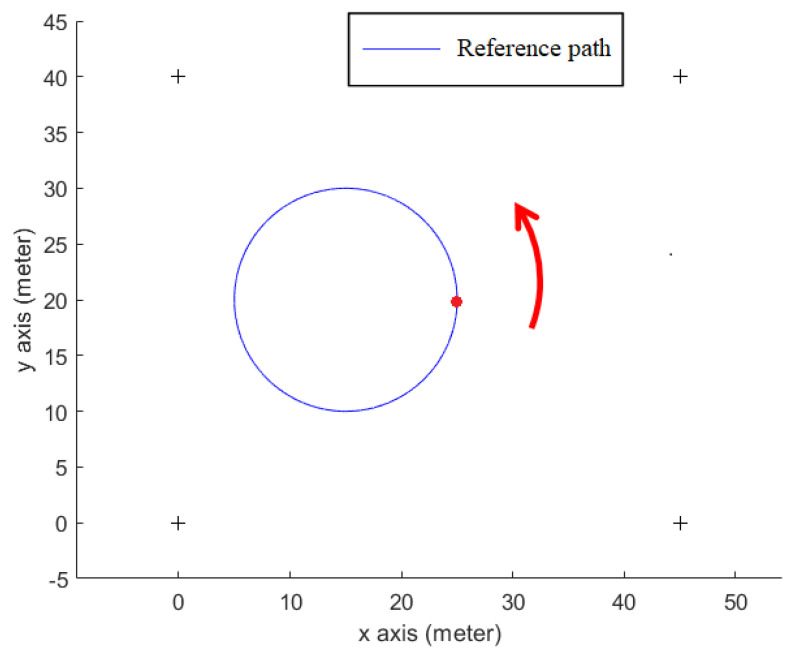
A circle path for the indoor positioning test.

As shown in Figure 7, a prearranged circle path (simulation data) within an indoor area (length: 45 m × width: 40 m), which has 4 tagged sound sources (+) allocated in the four corners of this area, is used. The radius of this prearranged circle path is 10 m, and the initial point of the receiver is at the point (*x_r_* = 25 me, *y_r_* = 20 m). In Figure 7, the mobile phone user (red dot) walks counterclockwise along the circle path (arrow).

**Figure 8:**
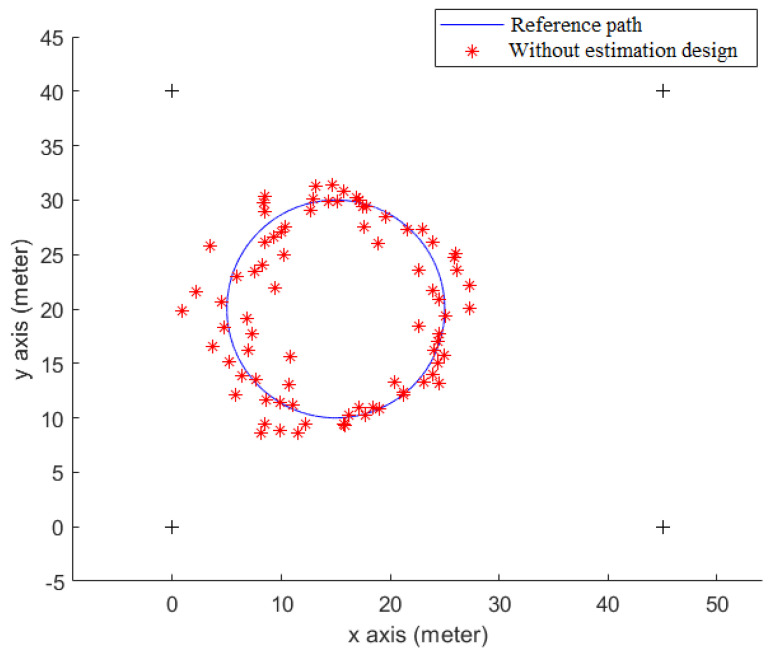
The positioning result without using any estimation design.

**Figure 9:**
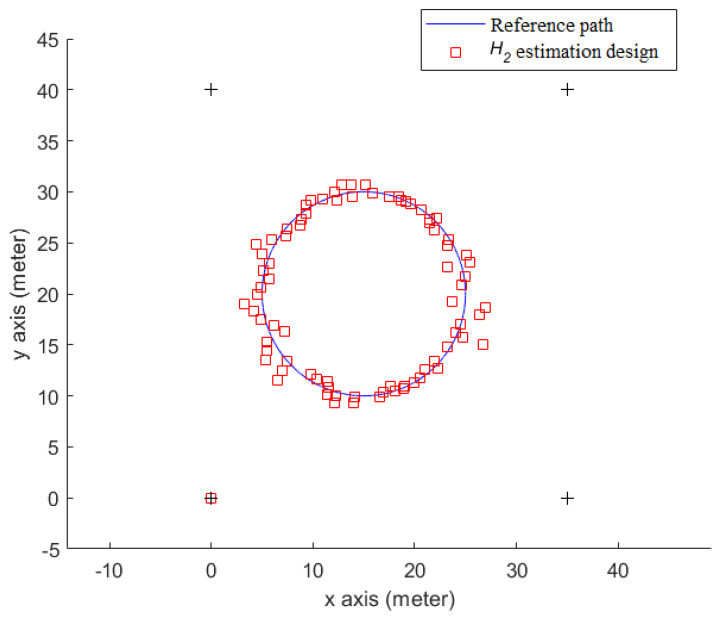
The indoor positioning result of the proposed *H*_2_ estimation design (simulation).

Figure 8 shows the indoor positioning result, which only uses measured SPLs and Equations (6) and (20) to calculate the user’s position (*x_r_*, *y_r_*), without the help of an estimation design. The root mean square (RMS) error of this simulation is 1.59 m. As for the positioning result of utilizing the *H*_2_ estimation design, it is plotted in Figure 9, and the RMS error is 0.77 m.

**Figure 10:**
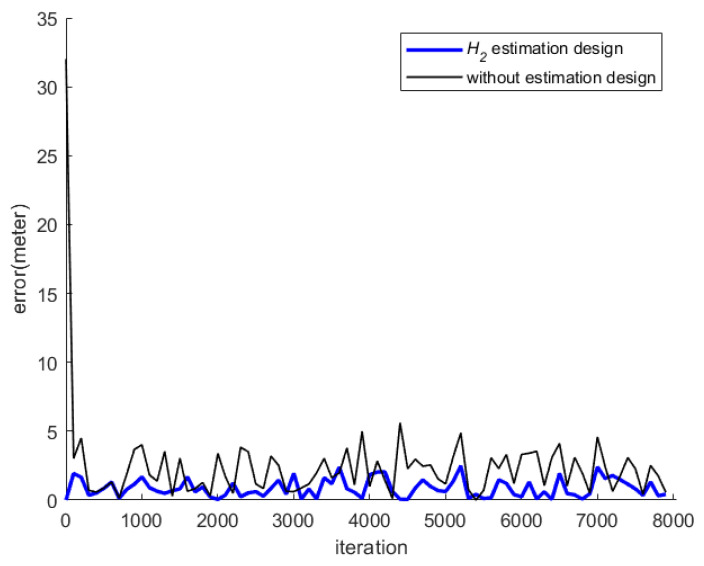
Histories of positioning errors of the proposed estimation design with respect to the positioning scheme without using any estimation design (simulation).

From Figure 9 and Figure 10, it is obvious that the indoor positioning accuracy can be effectively improved by the proposed estimation design, and the indoor positioning performance of the proposed method is superior to that without any estimation design.

### 3.3. Practical Experiments

**Figure 11:**
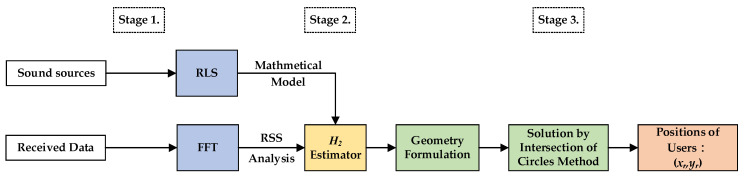
The flow chart of the proposed indoor positioning algorithm for practical tests.

Figure 11 shows the overall testing process of our proposed indoor positioning design. Similarly, this process for practical tests is also divided into three stages. In the first stage, the real time measured SPL data is used to build the mathematical walking model of the mobile phone user, and an RSS analysis using the FFT is adopted for identifying the first two tagged sound sources of the allocated four tagged sound sources. In stage 2, purified SPLs can be delivered, and in the third stage, real time positions of the mobile phone user can be solved by using the intersection of circles method.

**Figure 12:**
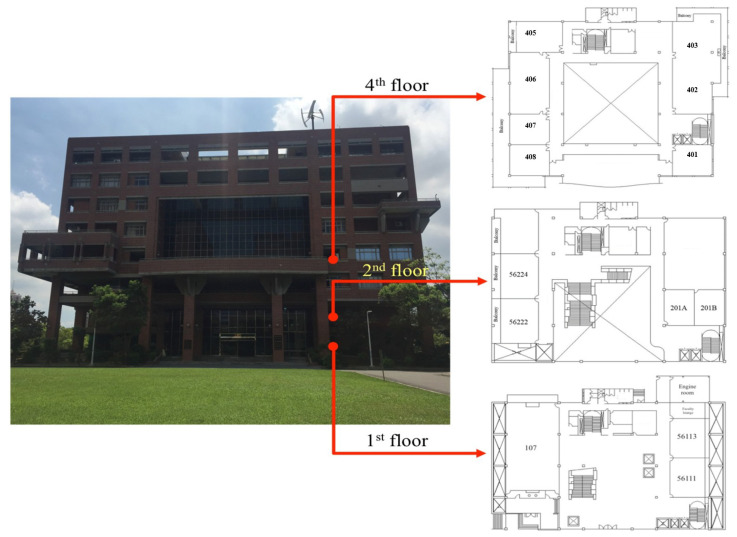
The test building and the layouts of floors for this proposed estimation design.

The practical experiment of this proposed design is conducted in the building of the Department of Systems and Naval Mechatronic Engineering, National Cheng Kung University, No.1, University Road, Tainan City, Taiwan. The tested building and layouts of the floors are shown as Figure 12. The iPhone6 is chosen as a receiver, and four tagged sound sources are allocated in appropriated positions, according to the requirements of the experiments. The sampling frequency of the received data is 44,100 Hz. Four distinct frequencies: 15.5 kHz, 16.5 kHz, 17.5 kHz, and 18.5 kHz, are selected for the purpose of identifying the detected sound sources. After the above arrangements in the test environment and apparatus, the same test pattern—a circle path—is assigned beforehand to verify the indoor positioning abilities of this proposed estimation design and the scheme without any estimation design.

**Figure 13:**
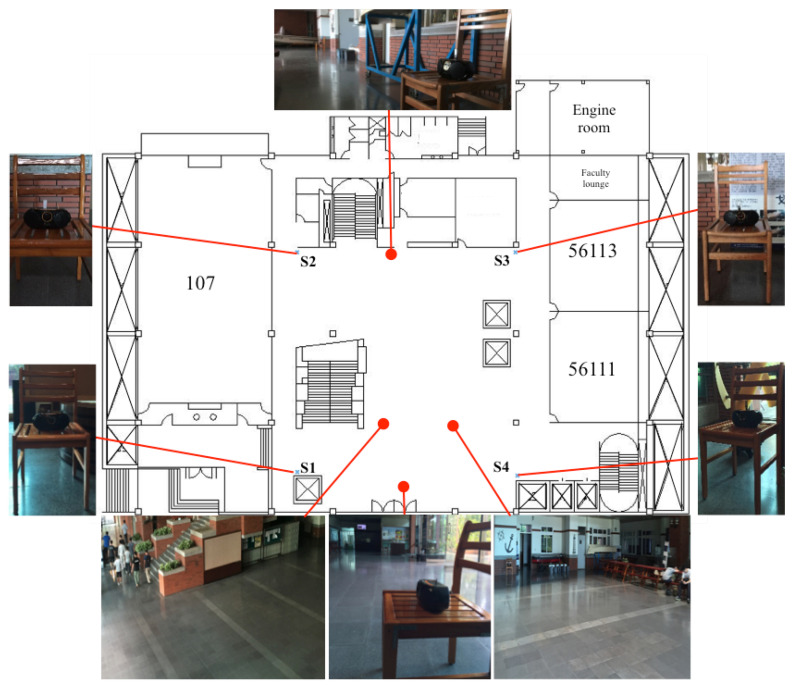
The test environment in the first floor of the building.

**Figure 14:**
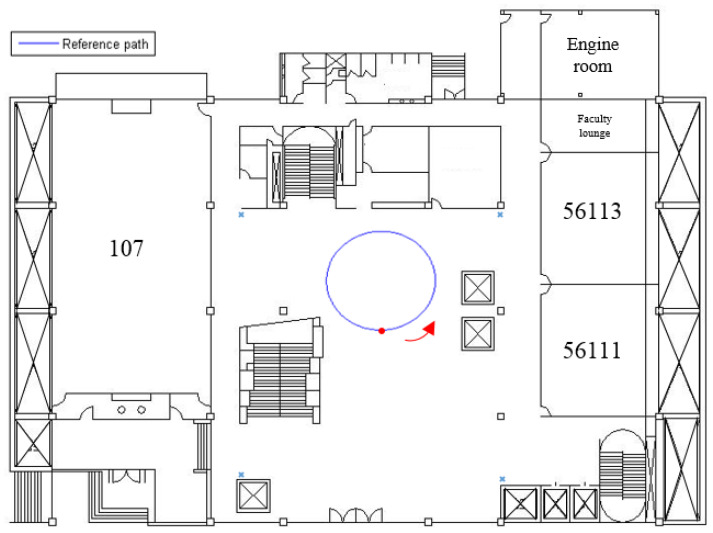
The circle path for the indoor positioning test in an indoor plane.

This real test is conducted in an area with dimensions: (40 m × 45 m) and covered with four tagged sound sources. The relative distance between the tagged sound sources (S1 to S4) is 18 m, as shown in Figure 13, and the cross stars represent the locations of the tagged sound sources. In this scenario, the test path has a circle trajectory, with a radius of 5 m. A total of 7998 SPL data sampled within 40 s are acquired in this test, and each real-time indoor positioning point is drawn per 0.01 Hz. The mobile phone user will walk counterclockwise along this circle path, as illustrated in Figure 14.

**Figure 15:**
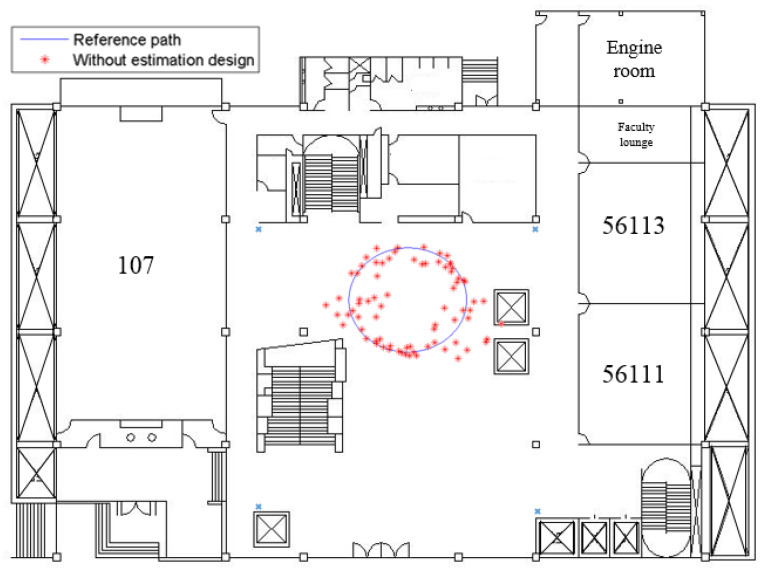
The indoor positioning result without any estimation design.

**Figure 16:**
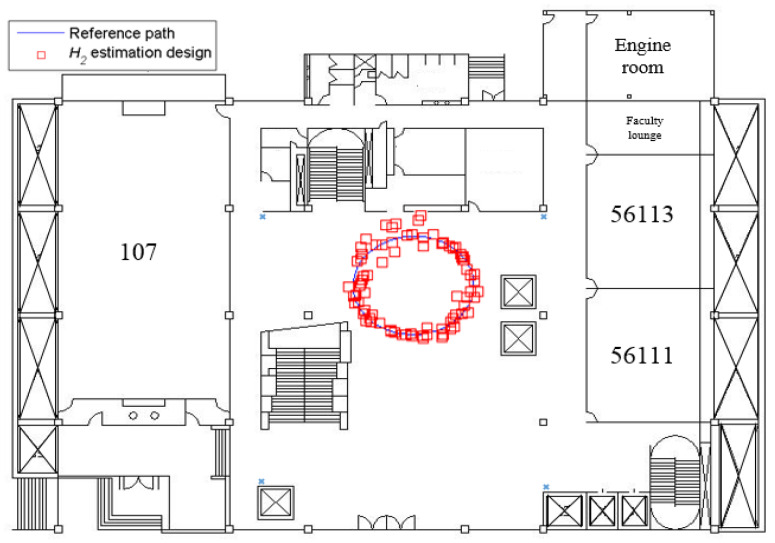
The indoor positioning result of the proposed *H*_2_ estimation design (real test).

**Figure 17:**
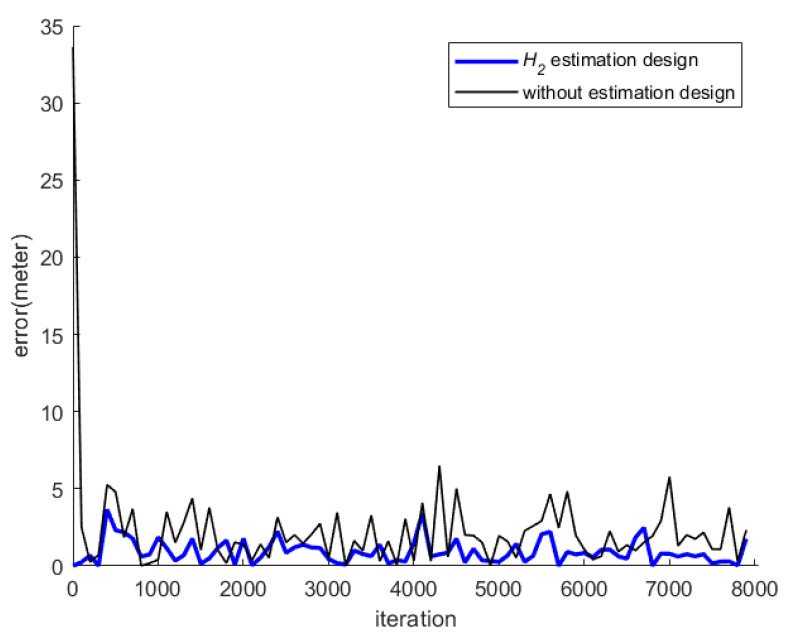
Histories of positioning errors of the proposed estimation design with respect to the positioning scheme without using any estimation design (real test).

Figure 15 shows the total indoor positioning result, without using any estimation design, and the RMS error with respect to the circle reference path is 2.49 m. Figure 16 shows the positioning result of the proposed *H*_2_ estimation design, and the RMS error, which corresponds to the circle reference path, is 0.74 m. Figure 17 shows the indoor positioning error histories of the proposed estimation design with respect to the indoor positioning scheme without any estimation design.

From Figure 16 and Figure 17, it can be seen that the proposed indoor positioning design highly improves the positioning accuracy, even under the effect of the worst background noises, and it outperforms the design that uses only measured SPLs, without any estimation design.

### 3.4. Comparisons of Simulation Results and Practical Results

As shown in Table 5, the RMS error of the indoor positioning result, without the help of estimation designs, is 1.59 m within an area with a length of 45 m and a width of 40 m.

As shown in Table 6, the RMS error of the indoor positioning performance of the *H*_2_ estimation design is 0.77 m, based on the same test environment and condition. 

In real experiments, the covered areas are similar to those of simulations. Table 7 shows the RMS error of the indoor positioning, without using the estimation design, within an area with a length of 45 m and a width of 40 m. 

As for the *H*_2_ estimation design, the positioning performance is listed in Table 8. Under the same test condition, the proposed *H*_2_ estimation design delivers an indoor positioning performance, with an RMS error of 0.77 m.

From comparisons of Table 5, Table 6, Table 7 and Table 8, it can be seen that the indoor positioning performances of this proposed method in simulation and practical experiments are similar because the noise levels of the environmental corruptions simulated by Equation (8) have been tuned beforehand, according to off-line measured environmental corruptions for the purpose of approximating the noise level of the true corruption in the real environment. Similar results can be obtained for the indoor positioning scheme without any estimation design. As a whole, the proposed indoor positioning design yields better positioning performance than that without any estimation design, and it is robust in regards to the environmental background noises.

## 4. Conclusions

An effective and accurate indoor positioning function is necessary for users who carry mobile phones because they spend a lot of time in shielding spaces. For achieving this design target, an indoor positioning scheme with the *H*_2_ estimation design, which can purify the corrupted SPLs of measured sound sources, is successfully developed for mobile phone users in this investigation. Although the test conditions vary randomly, and indoor environments are challenging due to the unknown background noises, simulation results and practical tests obviously show the promising indoor positioning performance of this proposed method: an average positioning RMS error of 0.75 m can be obtained. Two main contributions can be summarized for this investigation: 1. A compact indoor positioning system, with a high indoor positioning accuracy and capable of execution on mobile phones, is developed because the proposed *H*_2_ estimation design possesses the following natural potentials: a low power consumption for computation and an easy-to-implement filter structure. 2. This investigation provides a positioning possibility other than GPS for use in commonly unshielded spaces, as well as indoor environments.

## Figures and Tables

**Table 1:** Specifications of the Philips AT10.

Total output power	3 W
Frequency response	60–20,000 Hz, ± 3 dB
Signal to noise ratio	>70 dBA
MP3 bit rate	32–320 kbps
Dimensions	239 × 104 × 127 mm
Speaker impedance	6 ohm

**Table 2:** Specifications of iPhone 6.

Chip	A8 chip with 64 bits
CPU	Dual-core 1.4 GHz Typhoon (ARM v8-based)
Number of microphones	Triple microphones, bottom, front, rear (only one could be arbitrarily used by the developer.)
Dimensions	138.1 × 67 × 6.9 mm

**Table 3:** Setting values of each tagged sound source.

Number n	Sound Source $S_{n}$	Frequency $f_{\mathrm{Sn}}$	Relative Distance $r_{Sn}(0)$	SPL Value $l_{Sn}(0)$
1	S1	15.5 kHz	0.1 m	111.5 dB
2	S2	16.5 kHz	0.1 m	117.5 dB
3	S3	17.5 kHz	0.1 m	114.9 dB
4	S4	18.5 kHz	0.1 m	116.1 dB

**Table 4:** Initial values of the proposed *H*_2_ estimation design with respect to the tagged sound source S1.

Variables	Definitions	Values
${\hat{L}}_{S1}$	Initialization of estimation states	$0_{m\times 1}$
$Q_{S1}^{+}$	Error covariance	$I_{m\times m}$
$T_{S1}$	System disturbance covariance	0.5
$R_{S1}$	Measurement noise covariance	0.01
J	Constant matrix	$I_{m\times m}$
$\varepsilon_{S1}$	Positive value	20
$F_{v}$	Positive definite weighting matrix	80
$F_{w}$	Positive definite weighting matrix	80
U	Positive definite weighting matrix	$I_{m\times m}$
The system steady state parameters ${\hat{\lambda }}_{S1}$ and the weighting matrices of *H*_2_ estimation design under steady-state conditions.		
${\hat{\lambda }}_{S1}=\left[\begin{array}{c} 0.2132\\ 0.1922\\ 0.1712\\ 0.1503\\ 0.1294\\ 0.1084\\ 0.0875\\ 0.0666\\ 0.0457\\ 0.0248\\ 0.0039\\ -0.017\\ -0.0379\\ -0.0587\\ -0.0796 \end{array}\right]$, $G_{S1}=\left[\begin{array}{c} 0.2106\\ 0.9824\\ 2.274e^{-4}\\ 2.026e^{-4}\\ 1.778e^{-4}\\ 1.531e^{-4}\\ 1.283e^{-4}\\ 1.035e^{-4}\\ 7.879e^{-5}\\ 5.405e^{-5}\\ 2.932e^{-5}\\ 4.609e^{-6}\\ -2.01e^{-5}\\ -4.479e^{-5}\\ -6.948e^{-5} \end{array}\right]$ $E_{S1}={\left[\begin{array}{ccccc} 0.01682 & 0 & \cdots & \cdots & 0\\ 0 & 0.01682 & \ddots & \ddots & \vdots \\ \vdots & \ddots & 0.01682 & \ddots & \vdots \\ \vdots & \ddots & \ddots & 0.01682 & 0\\ 0 & \cdots & \cdots & 0 & 0.01682 \end{array}\right]}_{15\times 15}$		

**Table 5:** The positioning results without estimation designs in the simulation.

Reference Path	Covered Area	RMS Error
Circle	45 m × 40 m	1.59 m

**Table 6:** The positioning results of the proposed *H*_2_ estimation design in the simulation.

Reference Path	Covered Area	RMS Error
Circle	45 m × 40 m	0.77 m

**Table 7:** The positioning results without any estimation design in the practical experiments.

Reference Path	Covered Area	RMS Error
Circle	45 m × 40 m	2.49 m

**Table 8:** The indoor positioning of the proposed *H*_2_ estimation design in the practical experiments.

Reference Path	Covered Area	RMS Error
Circle	40 m × 45 m	0.74 m

## Data Availability

Not applicable.

## Appendix A

From Equations (35) and (36), the estimation covariance can be further derived as

(A1) $$\begin{array}{l} E\left\{{\tilde{L}}_{Sn}\left(k+1\right){\tilde{L}}_{Sn}{\left(k+1\right)}^{T}\right\}\\ \;\;=E\left\{\begin{array}{l} \left[\left(\Psi_{Sn}-G_{Sn}\Omega \right)L_{Sn}\left(k\right)+\Lambda w_{Sn}\left(k\right)-G_{Sn}\Pi v_{Sn}\left(k\right)\right]\\ \cdot \left[{\tilde{L}}_{Sn}{\left(k\right)}^{T}{\left(\Psi_{Sn}-G_{Sn}\Omega \right)}^{T}+w_{Sn}{\left(k\right)}^{T}\Lambda^{T}-v_{Sn}{\left(k\right)}^{T}\Pi^{T}G_{Sn}^{T}\right] \end{array}\right\}\\ \;\;=E\left\{\begin{array}{l} \left(\Psi_{Sn}-G_{Sn}\Omega \right){\tilde{L}}_{Sn}\left(k\right){\tilde{L}}_{Sn}{\left(k\right)}^{T}{\left(\Psi_{Sn}-G_{Sn}\Omega \right)}^{T}\\ +\Lambda w_{Sn}\left(k\right)w_{Sn}{\left(k\right)}^{T}\Lambda^{T}+G_{Sn}\Pi v_{Sn}\left(k\right)v_{Sn}{\left(k\right)}^{T}\Pi^{T}G_{Sn}^{T} \end{array}\right\}\\ \;\;=\left(\Psi_{Sn}-G_{Sn}\Omega \right)E\left\{{\tilde{L}}_{Sn}\left(k\right){\tilde{L}}_{Sn}{\left(k\right)}^{T}\right\}{\left(\Psi_{Sn}-G_{Sn}\Omega \right)}^{T}\\ \;\;\;\;\;\;\;\;\;\;\;\;\;\;\;+\Lambda \Lambda^{T}+G_{Sn}\Pi \Pi^{T}G_{Sn}^{T} \end{array}$$

where $L_{Sn}\left(k\right)$, $w_{Sn}\left(k\right)$, and $v_{Sn}\left(k\right)$ are mutually orthogonal, and the covariance matrix $E\left\{w_{Sn}\left(k\right)w_{Sn}{\left(k\right)}^{T}\right\}=I_{m\times m}$ and $E\left\{v_{Sn}\left(k\right)v_{Sn}{\left(k\right)}^{T}\right\}=I_{m\times m}$ are assumed as the identity matrix. The other covariance matrix $E\left\{{\tilde{L}}_{Sn}\left(k\right){\tilde{L}}_{Sn}{\left(k\right)}^{T}\right\}$, at a steady state in practical design, is constant (i.e.,k→∞) and is represented as $E\left\{{\tilde{L}}_{Sn}\left(k\right){\tilde{L}}_{Sn}{\left(k\right)}^{T}\right\}=\Theta_{Sn}$.

Combining Equation (35) and Equation (A1), the mean-square error can be rewritten as:

(A2) $$\begin{array}{ll} X_{Sn} & =tr\left(JE\left\{{\tilde{L}}_{Sn}\left(k+1\right){\tilde{L}}_{Sn}{\left(k+1\right)}^{T}\right\}J^{T}\right)\\ & =tr\left(J\left[\begin{array}{l} \left(\Psi_{Sn}-G_{Sn}\Omega \right)\Theta_{Sn}{\left(\Psi_{Sn}-G_{Sn}\Omega \right)}^{T}\\ +\Lambda \Lambda^{T}+G_{Sn}\Pi \Pi^{T}G_{Sn}^{T} \end{array}\right]J^{T}\right)\\ & =tr\left(J\left[\begin{array}{l} \left(\Psi_{Sn}-G_{Sn}\Omega \right)\Theta_{Sn}{\left(\Psi_{Sn}-G_{Sn}\Omega \right)}^{T}-\Theta_{Sn}\\ +\Lambda \Lambda^{T}+G_{Sn}\Pi \Pi^{T}G_{Sn}^{T} \end{array}\right]J^{T}\right)\\ & \;\;+tr\left(J\Theta_{Sn}J^{T}\right) \end{array}$$

Equation (A2) shows that the mean-square error $X_{Sn}$ must have an upper bound as below:

(A3) $$X_{Sn}\leq tr\left(J\Theta_{Sn}J^{T}\right)$$

under the following inequality holds:

(A4) $$\left(\Psi_{Sn}-G_{Sn}\Omega \right)\Theta_{Sn}{\left(\Psi_{Sn}-G_{Sn}\Omega \right)}^{T}-\Theta_{Sn}+\Lambda \Lambda^{T}+G_{Sn}\Pi \Pi^{T}G_{Sn}^{T}<0$$

Given that $E_{Sn}=\Theta_{Sn}^{-1}$, multiplying both sides of Equation (A4) by $E_{Sn}$ and selecting $D_{Sn}=E_{Sn}G_{Sn}$, the Equation (A4) can be rewritten as:

(A5) $$\begin{array}{l} \left(E_{Sn}\Psi_{Sn}-D_{Sn}\Omega \right)E_{2Sn}^{-1}{\left(E_{Sn}\Psi_{Sn}-D_{Sn}\Omega \right)}^{-1}\\ \;\;\;\;\;\;\;-E_{Sn}+E_{Sn}\Lambda \Lambda^{T}E_{Sn}+D_{Sn}\Pi \Pi^{T}D_{Sn}^{T}<0 \end{array}$$

For acquiring solution $E_{Sn}$ in Equation (A5), the Schur complement is employing to Equation (A5) for finding out a positive solution $E_{Sn}$, yielding the following equivalent LMI form:

(A6) $$\left[\begin{array}{cccc} E_{Sn} & E_{Sn}\Lambda & D_{Sn}\Pi & \left(E_{Sn}\Psi_{Sn}-D_{Sn}\Omega \right)\\ \Lambda^{T}E_{Sn} & I & 0 & 0\\ \Pi^{T}D_{Sn}^{T} & 0 & I & 0\\ {\left(E_{Sn}\Psi_{Sn}-D_{Sn}\Omega \right)}^{T} & 0 & 0 & E_{Sn} \end{array}\right]>0$$

This is Equation (37), and the proof is completed.
